# Effects of soybean isoflavones on reproductive parameters in Chinese mini-pig boars

**DOI:** 10.1186/2049-1891-3-31

**Published:** 2012-10-29

**Authors:** Xiao-xue Yuan, Bin Zhang, Li-li Li, Chao-wu Xiao, Jue-xin Fan, Mei-mei Geng, Yu-long Yin

**Affiliations:** 1 Institute of Subtropical Agriculture, The Chinese Academy of Sciences, Key Lab of Process of Subtropical Agriculture, Changsha, 410125, China; 2 Graduate University of Chinese Academy of Sciences, Beijing, 100049, China; 3 Hunan Agricultural University, Changsha, 410128, China; 4 Nutrition Research Division, Food Directorate, Health Canada, Ottawa, Ontario, K1A0K9, Canada

**Keywords:** Soy isoflavones, Male reproductive function, Pigs

## Abstract

**Background:**

Soybean isoflavones are structurally similar to mammalian estrogens and therefore may act as estrogen agonists or antagonists. However, it has not been determined if they have any negative effects on reproductive parameters in male livestock. Therefore, the objective of this study was to evaluate the effects of soybean isoflavones on male reproduction using Chinese mini-pig boars as a model. Fifty Xiang boars were randomly divided into five groups and fed diets containing 0, 125, 250, or 500 ppm soybean isoflavones or 0.5 ppm diethylstilbestrol for 60 days.

**Results:**

Dietary supplementation with 250 ppm of soy isoflavones markedly increased the testis index (*P* < 0.05), fructose content (*P* < 0.05), and α-glycosidase content in testicular tissue (*P* < 0.01), as well as increased the number of viable germ cells (*P* < 0.01) and the level of Bcl-2 protein (*P* < 0.01). However, 500 ppm of soybean isoflavones significantly reduced both testis and epididymis indexes (*P* < 0.05) and lactate dehydrogenase levels (*P* < 0.01), as well as reduced serum LH and testosterone levels (*P* < 0.05). High levels of soybean isoflavones also increased malondialdehyde levels (*P* < 0.05), as well as increased the numbers of early and late apoptotic germ cells (*P* < 0.01) and the level of Bax proteins (*P* < 0.05) in the testis.

**Conclusions:**

The results of this study indicate that consumption of soy isoflavones at dietary levels up to 250 ppm did not adversely affect reproductive parameters in Chinese mini-pig boars whereas higher levels of soy isoflavones may adversely affect male reproduction.

## Background

Phytoestrogens, especially soy isoflavones, have been suggested to be therapeutic for a wide range of estrogen-dependent diseases such as breast cancer, adverse menopausal symptoms, cardiovascular disease and osteoporosis [1,2]. Isoflavones are structurally similar to mammalian endogenous estrogens [3], and thus may act as estrogen agonists or antagonists [4]. Interest in the health effects of soy isoflavones has increased dramatically during the past few years since the development of reproductive organs and endocrine function have been shown to be influenced by consumption of plant-derived estrogen-like molecules [5].

Animal studies, particularly those conducted with rodents, have shown that high dietary intakes of soy isoflavones adversely affects female reproductive and endocrine function. For example, soy isoflavones increased uterine and ovarian weights as well as serum gonadotropin and FSH levels in female mice [6]. Treatment of neonatal mice with genistein, one of the major soy isoflavones, caused abnormal estrous cycles, altered ovarian function, early reproductive senescence, and sub-fertility or infertility [7]. In addition, female mice exposed to soy isoflavones showed early onset of vaginal opening, strong irregularity in estrous cycles (persistent estrus) and profound histo-pathological alterations, such as multi-follicular ovaries, endometrial hypertrophy, and diffuse hyperplasia of the anterior pituitary [8]. Pubertal Sprague–Dawley rats exposed to soy isoflavones had extended estrous cycles [9].

Although most of the published studies on the effects of soy isoflavones have used rodents or primates as models and focused on the female reproductive system, there is also evidence that male reproduction may also be affected. Exposure of male rats to dietary soy isoflavones increased testosterone levels in the serum and testis [9], and delayed the growth and development of the testis and also induced structural changes in testicular tissues [10]. It has also been demonstrated that intake of high levels of soy foods or soy isoflavones was associated with lower sperm concentrations in humans [11].

Soy protein is one of the main sources of feed protein for livestock [12]. However, the effect of soy isoflavones on the reproductive performance of male livestock has not been determined. Therefore, the following study was conducted to determine the effects of soy isoflavones on male reproduction using Chinese mini-pig boars as a model.

## Methods

This experiment was carried out in accordance with the Chinese Guidelines for Animal Welfare and Experimental Protocol and approved by the Animal Care and Use Committee of The Chinese Academy of Sciences. Soy isoflavones (with a purity of 80%) were obtained from Hunan Wangzhonghua Biological Technical Company (Changsha, China).

### Animals and experimental treatments

Fifty healthy Chinese mini-pig boars, 28 days of age and with an average body weight of 2.7 kg, were obtained from a local mini-pig herd in Huanjiang (Guangxi, China). The boars were randomly assigned to one of five dietary treatments with 10 boars assigned to each treatment. The dietary treatments consisted of a corn-based basal diet (Control) or similar diets supplemented with 125 (L group), 250 (M group), or 500 ppm (H group) of soy isoflavones or 0.5 ppm of diethylstilbesterol (DES). All diets were formulated to meet the nutritional requirements of Chinese mini-pig boars (Table 1). The boars were individually housed in an environmentally controlled facility with hard plastic slatted flooring, and had free access to feed and drinking water.

**Table 1:** **Composition and nutrient levels of the basal diet** (% **air**-**dry basis**)

**Ingredients**	**Content**	**Nutrient levels**^**2**^	**Content**
Corn	62.0	Digestible energy (MJ/kg)	13.40
Fish meal	8.0	Crude protein	15.30
Whey permeate	5.0	Calcium	0.60
Rapeseed meal	8.0	Total phosphorus	0.67
Wheat bran	10.0	Lysine	0.79
Fat powder	3.0	Methionine	0.56
Premix^1^	4.0		

^1^Premix provided for 1 kg of complete diet: Cu as copper sulfate, 1000 mg; Fe as iron sulfate, 2000 mg; Se as sodium selenite, 8300 mg; Zn as zinc oxide, 3500 mg; Mn as manganese oxide, 1300 mg; vitamin D_3_, 52800 IU; vitamin A as retinyl acetate, 301000 IU; vitamin E as α-tocopherol, 742 IU; vitamin K as menadione sodium bisulfate, 71 mg; vitamin B_2_, 30 mg; calcium pantothenate, 540 mg; niacin, 1073 mg; and vitamin B_12_, 0.8 mg.

^2^Digestible energy was calculated while the other nutrient levels were chemically determined.

At the end of a 60-day feeding period, blood samples (10 mL) were collected by puncture of the jugular vein between 08:00 and 10:00 h following a 12-h period of feed deprivation to avoid a postprandial effect on serum metabolites [13,14]. Blood samples were immediately centrifuged at 3,000 x g for 10 min to obtain serum, which was stored at −20°C for 1 wk until analysis.

When blood sampling was completed, pigs were immediately anesthetized with sodium pentobarbital (50 mg/kg body weight) and killed by jugular puncture [15]. Ten samples (5g/sample) of testicular tissue were collected immediately after slaughter, snap-frozen in liquid nitrogen, and stored at −80°C until needed for analysis.

#### Measurement of testis and epididymis weights

Pigs were weighed just before slaughter. After slaughter, the testes, including the epididymis, were removed by open surgical castration. After being washed with pre-warmed PBS (pH 7.4, 37°C), the surrounding tissues were trimmed from the testis and epididymis using sterile scissors and the epididymis was carefully removed from the testis. The weights of both the testis and epididymis were measured separately with a Sartorious Digital Balance (Precision Weighing Balances, Bradford, MA). A testicular index and epididymis index were determined using formulae from Franca et al. [16]. The formulae used were as follows:

$$\;\begin{array}{cc} \text{Testicular index}\;\left(\text{TI}\right)= & (\text{Bilateral testicle weight}\\ & /\text{total body weight})\times 100\% \end{array}$$

$$\;\begin{array}{cc} \text{Epididymis index}\;\left(\text{EI}\right) & =(\text{Bilateral epididymis weight}\\ & /\text{total body weight})\times 100\% \end{array}$$

#### Testicular biochemical analysis

Serum biochemical metabolites included fructose, α-glycosidase, lactate dehydrogenase, γ-glutamyl transferase enzyme and malondialdehyde. All assays were performed using a CX-4 Auto-Blood Biochemical Analyzer (Beckman Coulter, Brea, CA) according to the manufacturer’s instructions (Beijing Leadman Biochemistry Technology Company, Beijing, China).

#### Analysis of serum hormone concentrations

Gonadotrophin releasing hormone (GnRH), luteinizing hormone (LH), follicle-stimulating hormone (FSH), testosterone, estradiol and prolactin levels in serum were measured using Radioactive Immunoassay Kits from Tianjin Nine Tripods Biomedical Engineering (Tianjin, China).

#### Apoptosis assay by flow cytometry

The extent of apoptosis in spermatogenic cells was measured using an Annexin V-FITC Apoptosis Detection Kit (Beyotime Institute of Biotechnology, Jiangsu, China) following the manufacturer’s instructions.

#### Isolation of spermatogenic cells

The semineferous tubules were isolated from the left testis and sheared in pre-warmed PBS (pH 7.4, 37°C). Tissues were incubated with collagenase (0.5 mg/ml in PBS, pH 7.4) for 15 min at 33°C, and the mixture was centrifuged at 800 x g for 10 min. The supernatant was transferred to a new tube and mixed with PBS (pH 7.4) containing trypsinase (0.5mg/ml in water) and DNase I (1.0 μg/ml in water) and then incubated for 15 min at 33°C. At the end of digestion, samples were centrifuged at 1000 x g for 10 min and the supernatant was removed. Cells were re-suspended in 50 μl PBS containing 0.5% bovine serum albumin and filtered through a 150 μm mesh.

#### Detection of apoptosis

Before analysis, isolated cells were washed twice with PBS, gently re-suspended in AnnexinV Binding Buffer and incubated with AnnexinV-FITC/PI in the dark for 15 min and then analyzed by flow cytometry using Cell Quest Software (BD Biosciences, San Jose, CA). The fraction of the cell population in the different quadrants was analyzed using quadrant statistics. The dual parametric dot plots combining Annexin V-FITC and PI Fluorescence showed the viable cell population in the lower left quadrant (Annexin V^-^ PI^-^), the early apoptotic cells in the lower right quadrant (Annexin V^+^ PI^-^ ), and the late apoptotic cells in the upper right quadrant (Annexin V^+^ PI^+^ ) [17].

#### Western blot analysis

Tissue samples were homogenized in RIPA lysis buffer (150 mM NaCl, 1% Triton X-100, 0.5% sodium deoxycholate, 0.1% SDS, 50 mM Tris–HCl at pH 7.4), followed by centrifugation at 10 000 x g for 20 min. The total protein concentration in the supernatant was determined with the Bicinchoninic Acid Assay (Beyotime Biotechnology, Jiangsu, China). The protein was separated by a SDS–PAGE and electrotransferred to a nitrocellulose membrane. Immunostaining was conducted using specific antibodies against Bcl-2, Bax, and β-actin (Santa Cruz Biotechnology, Santa Cruz, Ca) following the procedures of An et al. [18], and the signals were detected by the enhanced chemiluminescence kit (Applygen Technologies, Beijing, China). AlphaImager 2200 software (Alpha Innotech Corporation, San Leandro, CA) was used to determine the density of the protein bands.

#### Chemical analysis of diets

The contents of dry matter, crude protein, and gross energy level in the diets were determined according to AOAC [19] methods. Amino acids in the diet were analyzed by HPLC [20].

#### Statistical analysis

Results are expressed as mean ± SEM. The effect of treatment was analyzed by a One-Way ANOVA using SPSS 13.0 (SPSS Inc., Chicago, IL). The significance of differences between individual means was determined by Duncan’s Multiple Range Test and were considered significant at *P* < 0.05.

## Results

### Testis index and epididymis index

The testis index in the pigs fed 500 ppm of isoflavones was 40% lower than that for the control group (*P* < 0.05), and 58% lower than in boars fed 250 ppm isoflavones (*P* < 0.01) and 51% lower than boars fed diethylstilbesterol (*P* < 0.01) (Table 2). The testis index in boars fed 250 ppm isoflavones was higher than in the control boars (*P* < 0.05), while there was no difference between boars fed the control, 125 ppm isoflavones or diethylstilbesterol. The epididymis index in the pigs fed 500 ppm of isoflavones was significantly lower than in boars fed the other treatments (*P* < 0.05).

**Table 2:** **Testis and epididymis index of Chinese mini**-**pig boars fed soybean isoflavones or diethylstilbestrol**

**Treatment**	**Level** (**ppm**)	**Testis index**	**Epididymis index**
Control	0 (C)	0.25 ± 0.02^b^	0.08 ± 0.01^a^
Soy isoflavones (ppm)	125 (L)	0.30 ± 0.04^ab^	0.08 ± 0.01^a^
	250 (M)	0.36 ± 0.03^a^	0.09 ± 0.01^a^
	500 (H)	0.15 ± 0.02^c^	0.06 ± 0.01^b^
Diethylstilbestrol	0.5	0.31 ± 0.03^ab^	0.09 ± 0.01^a^

Note: Means with different letters within a column differ (*P*<0.05).

### Testicular biochemical measurements

The effects of soy isoflavones on testicular tissue biochemical indicators in Chinese mini-pig boars is shown in Table 3. Dietary supplementation with soy isoflavones did not affect serum concentrations of γ-glutamyl transferase (*P* > 0.05). Serum concentrations of α-glycosidase in boars fed 250 and 500 ppm isoflavones as well as those fed diethylstilbesterol were significantly higher than in the control (*P* < 0.01), while α-glycosidase in boars fed 125 ppm isoflavones was 44% higher than in boars fed the control. The fructose content in boars fed 250 ppm isoflavones was 37% higher than for control boars (*P* < 0.05), while there was no difference between the control boars and boars fed 125 or 500 ppm isoflavones or diethylstilbesterol. The malondialdehyde level in boars fed 500 ppm isoflavones was 39% higher than in the control boars (*P* < 0.05), while there was no difference between the control boars and boars fed 125 or 250 ppm isoflavones or diethylstilbesterol. Serum concentrations of lactate dehydrogenase in boars fed 500 ppm isoflavones was 65% lower than in the control (*P* < 0.01), whereas lactate dehydrogenase in boars fed 250 ppm isoflavones was 24% higher than in boars fed diethylstilbesterol with neither treatment different from the control.

**Table 3:** **Effects of soybean isoflavones on testis biochemical indices of male Chinese mini**-**pig boars**

**Treatment**	**Level** (**ppm**)	**γ**-**Glutamyl Transferase** (**U**/**L**)	**Lactate Dehydrogenase** (**U**/**L**)	**α**-**Glycosidase** (**U**/**L**)	**Fructose** (**μmol·mg** /**pro**)	**Malondialdehyde** ( **nmol·mg** /**pro**)
Control	0 (C)	1095 ± 106	767 ± 109^ab^	18.4 ± 1.7 ^c^	204 ± 7.5^b^	8.9 ± 0.3^b^
Soy isoflavones (ppm)	125 (L)	963 ± 117	396 ± 171^bc^	33.2 ± 2.9 ^b^	221 ± 3.4^ab^	10.3 ± 0.9^ab^
	250 (M)	949 ± 244	1017 ± 236^a^	46.5 ± 0.5 ^a^	281 ± 4.5^a^	9.8 ± 0.7^b^
	500 (H)	1238 ± 148	270 ± 149^c^	44.1 ± 0.6 ^a^	249 ± 3.7^ab^	12.3 ± 0.7^a^
Diethylstilbestrol	0.5	942 ± 231	768 ± 75^ab^	48.2 ± 1.7 ^a^	229 ± 1.9^ab^	9.5 ± 0.9^b^

Note: Means with different letters within a column differ (*P*<0.05).

### Analysis of serum hormone concentrations

Serum concentrations of GnRH in boars fed 250 ppm isoflavones was 18% higher than in boars fed 500 ppm isoflavones (*P* < 0.05, Table 4). LH levels in boars fed 500 ppm isoflavones was 42% lower than in the control boars (*P* < 0.05). Dietary soy isoflavones supplementation did not affect serum concentrations of FSH or prolactin (*P* > 0.05). Serum concentrations of testosterone in boars fed 500 ppm isoflavones were 69% lower than in boars fed 250 ppm isoflavones (*P* < 0.01), 57% lower than in boars fed 125 ppm isoflavones (*P* < 0.05) and 54% lower than in the control boars (*P* < 0.05). In addition, serum concentrations of estradiol in boars fed 500 ppm isoflavones were 77% greater (*P* < 0.01) than in boars fed 250 ppm isoflavones, 67% greater (*P* < 0.05) than in boars fed 125 ppm isoflavones, 39% greater (*P* < 0.05) than in the control boars and 36% higher (*P* < 0.05) than in boars fed diethylstilbesterol (Table 4).

**Table 4:** **Concentrations of serum reproductive hormones in Chinese mini**-**pig boars fed soy isoflavones or diethylstilbestrol**

**Treatment**	**Level**	**GnRH**	**LH**	**FSH**	**Testosterone**	**Estradiol**	**Prolactin**
Control	0 (C)	0.67 ± 0.01^ab^	3.92 ± 0 .44^a^	2.80 ± 0.15	848 ± 50^b^	350 ± 46^b^	0.32 ± 0.28
Soy isoflavones (ppm)	125 (L)	0.64 ± 0.03^ab^	3.81 ± 0.44^a^	3.13 ±0 .25	920 ± 110^b^	187 ± 32^c^	0.60 ± 0.33
	250 (M)	0.72 ± 0.02^a^	3.54 ± 0.48^ab^	3.73 ± 0.47	1288 ± 84^a^	133 ± 18^c^	0.84 ± 0.25
	500 (H)	0.59 ± 0.03^b^	2.28 ± 0.27^b^	3.51 ± 0.51	392 ± 2 2^c^	578 ± 34^a^	0.57 ± 0.28
Diethylstilbestrol	0.5	0.66 ± 0.03^ab^	3.00 ± 0.43^ab^	3.49 ± 0.39	700 ± 75^b^	368 ± 45^b^	0.24 ± 0.17

Note: Means with different letters within a column differ (*P*<0.05).

### Apoptosis of spermatogenic cells

**Figure 1:**
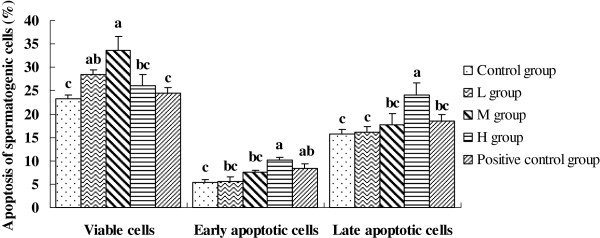
**Effects of different treatments on apoptosis of spermatogenic cells (%) of Chinese mini-pig boars fed diets containing 0 (Control), 125 (L), 250 (M), or 500 (H) ppm of soy isoflavones or 0.5 ppm diethylstilbesterol for 60 days.** Values are means ± SEM, n = 10, Means with different letters differ (P < 0.05).

The number of viable cells in the testicles of boars fed 250 ppm isoflavones were 31% higher than in the control boars (*P* < 0.01), and 27% higher than in boars fed diethylstilbesterol (*P* < 0.01). The early apoptotic cells in the testicles of boars fed 500 ppm isoflavones were 47% higher than in the control boars (*P* < 0.01). In addition, the late apoptotic cells in the testicles of boars fed 500 ppm isoflavones were 35% higher than in the control boars (P < 0.01), and 33% higher than in boars fed diethylstilbesterol (*P* < 0.01, Figure. 1).

### Western blot analysis of Bcl-2 and Bax contents in testicular tissue

**Figure 2:**
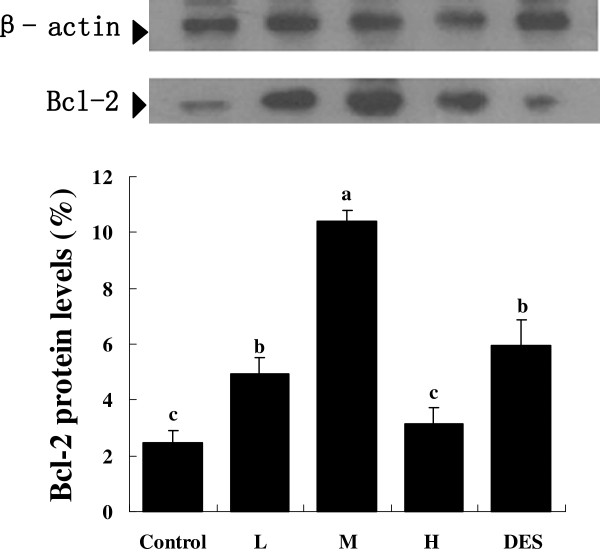
**Testicular Bcl-2 protein content in the Chinese mini-pig boars fed diets containing 0 (Cl), 125 (L), 250 (M), or 500 (H) ppm of soy isoflavones or 0.5 ppm diethylstilbesterol for 60 days.** The Western Blot image shown is representative of five replicates. Values are means ± SEM, n =10. Means with different letters differ (*P*<0.05).

**Figure 3:**
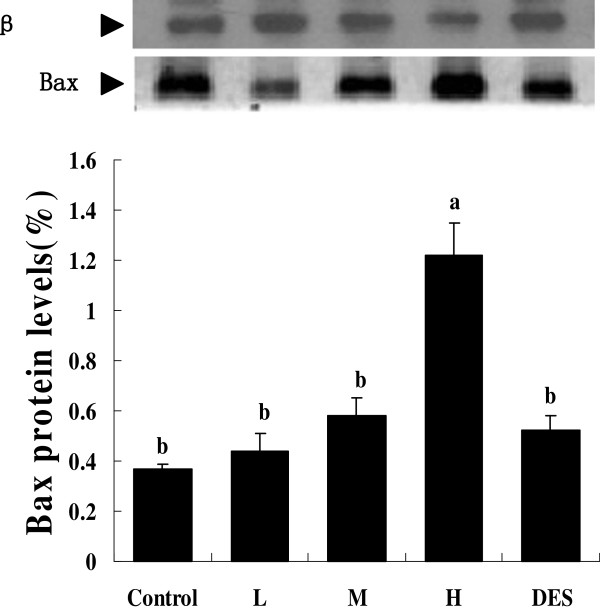
**Testicular BAX protein content in the Chinese mini-pigs fed diets containing 0 (C), 125 (L), 250 (M), or 500 (H) ppm of soy isoflavones or 0.5 ppm diethylstilbesterol for 60 days.** The Western Blot image shown is representative of five replicates. Values are means ± SEM, n =10. Means with different letters differ (*P*<0.05).

The Bcl-2 protein levels in the boars fed 125 ppm isoflavones, 250 ppm isoflavones or diethylstilbesterol were significantly higher than in boars fed the control or 500 ppm isoflavones (*P* < 0.05) (Figure 2). The pigs fed 500 ppm isoflavones had significantly higher testicular Bax protein content than boars fed 0, 125 or 20 ppm isoflavones or diethylstilbesterol (*P* < 0.05, Figure 3).

## Discussion

Although isoflavones are one of the most studied components in soy, their influence on reproductive functions is still not fully understood. In particular, their potential impact on male reproduction in non-rodent species remains to be determined. This study used male Chinese mini-pig boars as an experimental model to examine the effect of soy isoflavones on the male reproductive system by measuring their testis and epididymis index, testicular tissue biochemical indicators, serum hormones, apoptosis of spermatogenic cells, and related protein expression in testicular tissue.

It would appear that diethylstilbesterol had few significant effects on the parameters measured compared with the control in this study. A potential explanation for this is that the concentration used was not high enough to induce any negative effects on the parameters measured.

The testis and epididymis are important components of the male reproductive system. The testis is mainly composed of spermatogenic cells, leydig cells and sertoli cells. It is the place where germ cells mature from spermatogonium to spermatozoon. The epididymis is the transmission channel and storage space for sperm and is also the location where immature sperm change into mature sperm with mobility and fertilization ability [21].

In this study, we observed that 250 ppm soy isoflavones increased testicular weight while 500 ppm decreased the weight of the testis and epididymis in comparison with the control diet. This is consistent with what had been found in other studies [10,22,23]. Han et al. [24] demonstrated that the testicular weight increased in sexually mature male mice fed 100 ppm soy isoflavones. This might be attributed to the role of soy isoflavones in the growth axis, which increased growth hormone levels and increased the liver growth hormone receptors, so as to promote animal growth [25]. This stimulatory effect might be also due to the estrogenic actions of soy isoflavones. Through binding to estrogen receptors in the hypothalamus, pituitary gland, and other reproductive organs, soy isoflavones have been shown to facilitate the production and release of testosterone in male animals [26], which subsequently stimulated spermatogenesis, sperm maturation and growth of the testis.

Previous studies found that male reproductive function can be changed by all kinds of factors in experimental animals, which were usually accompanied by anomalous testicular biochemical function. It is very important to ensure the stability of the environment for spermatogenesis by maintaining a normal testicular biochemical state. Therefore, this study analyzed the changes in testicular biochemical metabolism to help understand the function of the testicles.

Fructose is regarded as the main source of sperm energy [27]. Previous research has shown that fructose increased the proportion of linear motioning sperm and induced the proportion of swinging sperm [28]. In this study, soy isoflavones increased fructose content in boars fed 250 ppm isoflavones compared with control boars. This demonstrates that supplementation with 250 ppm isoflavones ensured sufficient energy for the sperm which would improve sperm quality in Chinese mini-pig boars.

This study found that supplementation with soy isoflavones increased α-glycosidase the conten in testicular tissue compared with control boars. This suggests that soy isoflavones increased the activity of α-glycosidase, which provided energy for sperm metabolism and improved sperm quality.

Lactate dehydrogenase exists widely in sperm cells. Lactate dehydrogenase turns pyruvic acid into lactic acid to ferment without oxygen [29]. Lactate dehydrogenase levels in boars fed 500 ppm isoflavones were lower than in control boars. Moreover, this study showed that malondialdehyde levels in boars fed 500 ppm isoflavones was increased in testicular tissue compared with control boars which suggests that supplementation with 500 ppm soy isoflavones increased the degree of testicular lipid peroxidation. Our results are consistent with Zhou et al. [30] in rats.

Supplementation with 500 ppm isoflavones significantly decreased LH and testosterone levels compared with control Chinese mini-pig boars. This is consistent with the results reported by Hales et al. [31]. We assume that soy isoflavones affect the production and release of testosterone through regulation of the endocrine axis. The possible mechanism involved might be through affecting the secretion of androgen and the interaction of LH and LH receptors, which reduced testosterone levels in Leydig cells stimulated by LH. In this experiment, testosterone and estradiol levels in boars fed 250 and 500 ppm isoflavones were significantly different from the control boars. It is generally believed that soy isoflavones affect the estradiol feedback regulation in the hypothalamus-pituitary axis, and alter the endogenous testosterone level. Ma et al. [32] have demonstrated that soy isoflavones increased secretion of testosterone by reducing the transformation of testosterone to estradiol. The underlying mechanism might be associated with the ability of soy isoflavones to activate the cAMP/PKA signal pathways in Leydig cells, which leads to suppressed activity of aromatase.

In the testis of adult animals, the number of mature sperm was 20 to 75% lower than expected [33,34], indicating that a large number of germ cells diminished in the process of proliferation and maturation. Apoptosis is shown to be the main mechanism responsible for the reduction of germ cells. Besides spontaneous apoptosis, many factors can induce germ cell apoptosis in experimental animals, including hormone withdrawal, heat stimulation of testis, exposure to radiation [35], deficiency of trace elements [36], stress [37], and toxic materials [38,39]. To the best of our knowledge, there is no information available on whether consumption of soy isoflavones induces germ cell apoptosis in pigs.

The present study showed that dietary supplementation with 500 ppm isoflavones significantly increased the early apoptotic and late apoptotic germ cells in the testis compared with control boars and boars fed 125 and 250 ppm isoflavones. However, the boars fed 250 ppm isoflavones had significantly more viable germ cells in the testis. However, viable germ cells in boars fed 125 ppm isoflavones were not statistically different from boars fed 250 or 500 ppm isoflavones. In addition, our data showed that Bcl-2 protein levels in boars fed 250 ppm isoflavones was higher than in the control boars, which is consistent with the result of apoptosis measured by FCM (Flow Cytometry) in our research (Figure 2).

In boars fed 500 ppm isoflavones, the Bcl-2 content was lower than in boars fed 125, or 250 ppm isoflavones or diethylstilbesterol, whereas Bax, a proapoptotic protein, was higher than in all the other treatments. This was also consistent with increased germ cell apoptosis in boars fed 500 ppm isoflavone.

Bcl-2 and Bax are two important proteins involved in apoptosis, and the ratio of Bcl-2 and Bax in the cells is an indicator of apoptosis [40]. Treatment with radiation [41], or 2-bromopropane [42], or testosterone withdrawal [43] significantly changed the ratio of Bcl-2 and Bax in the cells. Alteration of the ratio of Bcl-2 to Bax in the germ cells might be one of the mechanisms by which the high dose of soy isoflavones induces germ cell apoptosis in Chinese mini-pig boars.

## Conclusions

In conclusion, our study showed that dietary supplementation with lower amounts of soy isoflavones (250 ppm) markedly increased the testis index, as well as fructose, α-glycosidase, viable germ cells and Bcl-2 protein levels in testicular tissue. However, higher amounts of isoflavones (500 ppm) significantly reduced testis and epididymis indexes, lactate dehydrogenase, serum LH and testosterone levels, and increased malondialdehyde contents, testicular Bax protein content as well as the numbers of early and late apoptotic germ cells in the testis in male Chinese mini-pig boars. Overall, our results suggest that consumption of lower amounts of soy isoflavones (up to 250 ppm) have no adverse effects on reproductive parameters, whereas higher amounts (500 ppm) of soy isoflavones may negatively affect male reproductive function.

## Abbreviations

DES: diethylstilbene; LDH: lactate dehydrogenase; GGT: γ-glutamyl transferase enzyme; MDA: Malondialdehyde; GnRH: gonadotrophin releasing hormone; LH: luteinizing hormone; FSH: follicle-stimulating hormone; Tes: testosterone; E_2_: estradiol; PRL: prolactin; Bcl-2: B-cell lymphoma 2; Bax: Bcl-2 Associated X protein.

## Competing interests

This publication does not mention trade names, commercial products or organizations which imply endorsement by the Chinese Government. Author disclosure: X.X Yuan, B. Zhang, L.L. Li, C.W. Xiao, J.X. Fan, M.M. Geng, and Y.L. Yin have no conflict of interest. This research was jointly supported by grants from the National Natural Science Foundation of China (No. 30972118) and Hunan Natural Science Foundation (No. 08JJ3080).

## Authors’ contributions

XXY, LLL, and CWX designed the research; XXY, JXF, and LLL conducted the research; XXY, and JXF analyzed the data; XXY, JXF, CWX, and LLL wrote the paper; and LLL had primary responsibility for the final content. All authors read and approved the final manuscript.
